# Noise-Induced Cochlear Damage Involves PPAR Down-Regulation through the Interplay between Oxidative Stress and Inflammation

**DOI:** 10.3390/antiox10081188

**Published:** 2021-07-26

**Authors:** Fabiola Paciello, Anna Pisani, Rolando Rolesi, Vincent Escarrat, Jacopo Galli, Gaetano Paludetti, Claudio Grassi, Diana Troiani, Anna Rita Fetoni

**Affiliations:** 1 Fondazione Policlinico Universitario A. Gemelli IRCCS, 00168 Roma, Italy; fabiola.paciello@unicatt.it (F.P.); jacopo.galli@unicatt.it (J.G.); gaetano.paludetti@unicatt.it (G.P.); 2 Department of Neuroscience, Università Cattolica del Sacro Cuore, 00168 Roma, Italy; diana.troiani@unicatt.it; 3 Department of Otolaryngology Head and Neck Surgery, Università Cattolica del Sacro Cuore, 00168 Roma, Italy; anna.pisani@unicatt.it (A.P.); rolandorolesi@gmail.com (R.R.); escarrat.v@outlook.fr (V.E.)

**Keywords:** acoustic trauma, cochlea, NF-κB, ROS, IL-1β, Q-ter, Anakinra

## Abstract

The cross-talk between oxidative stress and inflammation seems to play a key role in noise-induced hearing loss. Several studies have addressed the role of PPAR receptors in mediating antioxidant and anti-inflammatory effects and, although its protective activity has been demonstrated in several tissues, less is known about how PPARs could be involved in cochlear dysfunction induced by noise exposure. In this study, we used an in vivo model of noise-induced hearing loss to investigate how oxidative stress and inflammation participate in cochlear dysfunction through PPAR signaling pathways. Specifically, we found a progressive decrease in PPAR expression in the cochlea after acoustic trauma, paralleled by an increase in oxidative stress and inflammation. By comparing an antioxidant (Q-ter) and an anti-inflammatory (Anakinra) treatment, we demonstrated that oxidative stress is the primary element of damage in noise-induced cochlear injury and that increased inflammation can be considered a consequence of PPAR down-regulation induced by ROS production. Indeed, by decreasing oxidative stress, PPARs returned to control values, reactivating the negative control on inflammation in a feedback loop.

## 1. Introduction

Recent findings in hearing loss research have provided strong evidence for common predominant mechanisms of damage affecting the organ of Corti: the unbalance of cellular redox status and inflammation. The noise-induced reactive oxygen species (ROS) interact in many ways with the key transcription factor driving inflammation, the nuclear factor kappa-B (NF-κB) signaling pathway [1,2,3,4]. Indeed, noise exposure has been shown to up-regulate cochlear production of cytokines [5,6,7], such as interleukin 1-β (IL-1β), that have been observed after ROS generation in the cochlea [8]. Similar mechanisms have been reported to occur in drug-induced hearing loss [9,10] and aging [11].

We have previously reported that in animal models of exogenous insults as noise exposure or ototoxic drugs, it is possible to attenuate hearing loss and cochlear damage by targeting the endogenous antioxidant system, [10,12,13,14]. Indeed, we obtained significant ROS reduction and hearing loss amelioration by using antioxidants such as polyphenols and CoQ10 analogues [12,15,16,17]. Interestingly, we also showed that local delivery of pioglitazone, a PPARγ agonist, had potent anti-oxidant and anti-inflammatory effects in noise-induced hearing loss (NIHL), attenuating threshold shift through reduced NF-κB and IL-1β and decreasing superoxide and lipid peroxidation in cochlear samples [18].

PPARs comprise a family of three ligand-regulated transcription factors involved in many physiological and pathological processes [19,20,21,22,23]. Three major isoforms were identified and cloned so far includes, PPARα, PPAR-β/δ and PPAR-γ. They are expressed in different tissues and have central roles in the homeostasis and energy metabolism, regulating energy storage. PPARα is expressed highly in the liver, plays a role in fatty acid oxidation which provides energy for peripheral tissues, lipoprotein metabolism, and also has a potential role in oxidant/antioxidant pathway. PPAR-δ/β promotes fatty acid metabolism and suppresses macrophage-derived inflammation. PPARγ is highly expressed in adipose tissue, where it is a regulator of adipogenesis, lipid metabolism and insulin sensitivity [24]. Downstream of their metabolic effects, these agents modulate cellular oxidative stress through several pathways that regulate cellular oxidative balance, including the ROS production pathway and the Akt/Pi3K pathway [25,26,27]. PPAR agonists not only can act on oxidative stress and inflammatory pathways [21,28] but they can also activate antioxidant enzymes [29,30], also in the cochlea [31,32]. Interestingly, the expression of PPARs in the cochlea has been reported recently [32]. PPARγ and PPARα were found to be highly expressed in several cochlear cell types, including inner (IHCs) and outer (OHCs) hair cells. PPARs are able to regulate inflammatory processes in multiple organs by a direct interaction with NF-κB. In fact, PPARγ is able to regulate inflammation by a direct negative interaction with NF-κB [33].

Despite the growing interest in the protective effects of PPARs targeting against inflammatory and oxidative damage, the role of PPARs in mediating inflammation and oxidative stress in cochlear noise-induced damage has not been investigated. Thus, the aim of our study was to evaluate if and how acoustic trauma affects PPARγ and PPARα expression and what is the role of PPARs in the cross-talk between oxidative stress/inflammatory damage induced by noise. In addition, in order to investigate the roles of redox unbalance and inflammation in PPAR cochlear expression, we used an antioxidant (Q-ter, the water-soluble form of the endogenous antioxidant coenzyme Q10) to block oxidative stress and an anti-inflammatory (Anakinra, an antagonist of IL1 receptor) treatment as a tool to block inflammation. Our results indicate that noise-induced ROS overproduction down-regulates PPAR expression and, consequently, the over expression of inflammatory cytokines, which in turn will further increase ROS production though the induction of a vicious cycle.

## 2. Materials and Methods

### 2.1. Animals

**Figure 1:**
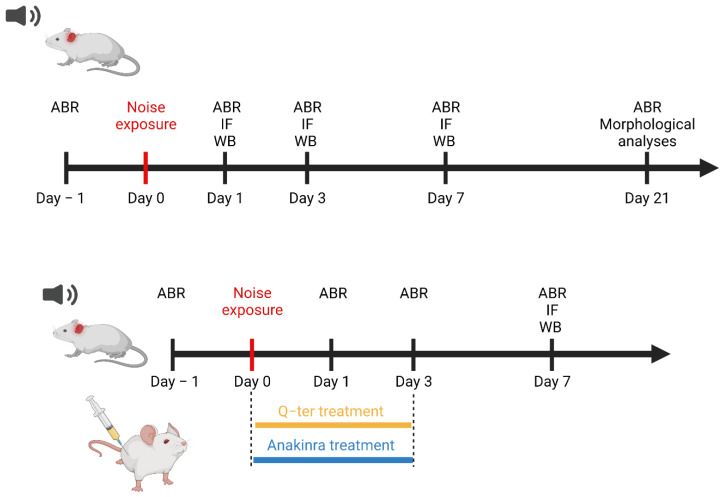
Experimental design and protocol. Male adult Wistar rats 2 months of age at the beginning of the study were used. Baseline hearing thresholds were evaluated the day before the exposure to noise (pure tone, 10 kHz, 120 dB SPL, 60 min). A cohort of animals was treated with Q-ter (100 mg/kg) or Anakinra (40 mg/kg) 1 h before and for the following 3 days after noise exposure. Functional, morphological and molecular (WB/IF) evaluations were performed at different time points after noise exposure (from day 1 to day 21). ABR: auditory brainstem response; IF: immunofluorescence; WB: Western immunoblot.

In this study, we used male adult Wistar rats (Catholic University Laboratories, 200–250 g) of 2 months of age, with normal Preyer’s reflex. The experiments were performed on a total of 96 animals, randomly assigned to 6 experimental groups as follows: (1) Control (“Ctrl” group; *n* = 24); (2) Control animals treated with Q-ter (“Ctrl-Q-ter” group; *n* = 6); (3) Control animals treated with Anakinra (“Ctrl-Anakinra” group; *n* = 6); (4) Noise-exposed animals (“Noise” group; *n* = 48) (5) Noise-exposed animals treated with Q-ter (“Noise+Q-ter” group; *n* = 6); and (6) Noise-exposed animals treated with Anakinra administration (“Noise+Anakinra” group; *n* = 6). In a preliminary study, 15 additional animals were used to determine the most protective dose of Anakinra (30, 40 and 50 mg/kg). Animals were sacrificed under deep anesthesia (ketamine at 70 mg/kg and medetomidine-domitor at 1 mg/kg) at days 1, 3, 7 and 21 after noise exposure. Experimental design and protocol timeline of experiments are summarized in Figure 1. For the whole experimental period, the animals were housed two per cage at controlled temperature (22–23 °C) and constant humidity (60 ± 5%), under a 12 h light/dark cycle, with food (Mucedola 4RF21, Italy) and water ad libitum.

### 2.2. Drug Administration

Q-ter is a terclatrate substance obtained by mechano-physical activation, a solid-state procedure that brings different substances into supramolecular contact through the administration of energy, and turning a simple mixture into a multi-composite material (Applicant, Asoltech Srl, Trieste, Italy). Q-ter consists of an outer case (an inactive pharmaceutical grade excipient) that entraps CoQ10 moieties (10% *w/w*) and an amino acid that serves as a catalyst to enable the formation of the multicomposite. Q-ter was provided by Scharper Therapeutics, Milan, Italy, and was manufactured using an industrially available native CoQ10 (Kaneka Pharma Europe, Brussels, Belgium). For the purposes of treatment, Q-ter was dissolved in saline and injected intraperitoneally (i.p.) at a dose of 100 mg/kg body weight (b.w.), based on previous studies [12,34].

Anakinra (Kineret) was provided by Swedish Orphan Biovitrum AB (publ) (Sobi™). For the purpose of the experiment, Anakinra was injected intramuscularly (i.m.) at a dose of 40 mg/kg, selected on the basis of the dose–response curve for different Anakinra doses. In order to evaluate the ability of Q-ter and Anakinra to rescue hearing loss, animals were treated 1 h before and the consecutive 3 days after acoustic trauma.

Animals treated with Q-ter (Ctrl-Q-ter) and Anakinra (Ctrl-Anakinra) alone did not show any significant change at ABR threshold with respect to the Ctrl group (data not shown). Therefore, immunostaining procedure was performed on the Ctrl, Noise, Noise+Q-ter and Noise+Anakinra groups.

### 2.3. Noise Exposure

As described previously [14,16], acute noise exposure was induced by a continuous pure tone of 120 dB SPL, centered on 10 kHz generated by a waveform generator (LAG-120B, Leader, New York, NY, USA) and amplified by an audio amplifier (A-307R, Pioneer, Tokyo, Japan). Under anesthesia (ketamine, 35 mg/kg and medetomidine-domitor, 0.25 mg/kg. i.m.), animals were placed in a soundproof room in a fixation cradle (their heads were gently maintained in a fixed position by a neck and a nose ring). The loud sound was presented for 60 min to the ears in free field by using loudspeakers (TW034x0, Audax, France) positioned 10 cm in front of the animal’s head. Sound level was measured using a calibrated 1/4 inch microphone (Model 7017, ACO Pacific Inc., Belmont, CA, USA) and a calibrated preamplifier (Acoustic Interface System, ACO Pacific Inc., Belmont, CA, USA).

### 2.4. Auditory Brainstem Responses (ABRs)

In order to assess hearing loss induced by noise exposure and the protective effects of Q-ter and Anakinra treatments, hearing function was estimated by ABR recordings. In all animals, ABRs were assessed at low (6 kHz), mid (12, 16 and 20 kHz) and high (24 and 32 kHz) frequencies before and at 1, 3, 7 and 21 days from noise exposure. ABR measurements were performed under mild anesthesia (ketamine, 35 mg/kg and medetomidine-domitor, 0.25 mg/kg, i.m.), positioning animals in the anechoic room. As described previously [14,16], 3 stainless steel recording electrodes were subcutaneously inserted posterior to the tested pinna (active), vertex (reference) and contralateral pinna (ground). ABR data acquisition and digital signal processing were performed by using a PC-controlled TDT System 3 (Tucker Davis Technologies, Alachua, FL, USA). Tone bursts of pure tones from 6 to 32 kHz (1 ms rise/fall time, 10 ms total duration, 20/s repetition rate) were presented monaurally. Responses were filtered (0.3–3 kHz), digitized and averaged (across 512 discrete samples at each frequency-level combination). The threshold value was defined as the lowest stimulus level that yielded a repeatable waveform-based onset.

### 2.5. F-Actin Staining: Hair Cell Count

In order to visualize hair cells and to quantify cell survival, we performed F-Actin staining on surface preparations of the organ of Corti was performed. At day 21 from noise exposure, after ABR recordings, animals were sacrificed with a lethal dose of anesthetic and the right cochleae of 6 animals/group were processed to perform IHC and OHC count. As described previously [10,14], to perform F-Actin staining, samples were fixed in 4% paraformaldehyde and incubated with Actin Green 488 Ready Probes Reagent (ThermoFisher, Waltham, MA, USA, Cat. No. R37110). All samples were mounted onto glass slides with a mounting medium (FluorSave TM Reagent, Merk, Darmstadt, Germany, Cat. No. 345789) and analyzed using a confocal microscope (Nikon Ti-E, Confocal Head A1 MP, Tokyo, Japan) equipped with an oil–immersion objective (40×). Hair cells were counted in segments of ~250 µm in length each along the basilar membrane. Hair cells were considered missing if both the stereocilia bundles and the cuticular plates were absent, and cell loss was calculated as percentage with respect to controls.

### 2.6. Hematoxylin and Eosin Staining: SGN Survival

To determine the noise effect on spiral ganglion neurons (SGNs) survival, 6 cochleae/group (from 6 animals) were processed at 21 days after noise exposure. As described previously [10], cochleae were quickly removed and the samples were fixed with 4% paraformaldehyde in PBS at 4 °C and at pH 7.5. Next, the cochleae were decalcified for 15 days in EDTA (10%), incubated for 48 h in sucrose (30%), embedded in OCT and longitudinal sections (12 μM thickness) were obtained by using a cryostat (Cryostat CM 1950; SLEE). Sections were stained with hematoxylin and eosin (H&E) to visualize spiral ganglion cells. Sections were incubated in hematoxylin (4–5 min) and in eosin (45 s), and then mounted with Entellan^®^ (Cat. No. 107960, Merck, Darmstadt, Germany). SGNs with a clear round nucleus and homogeneous cytoplasm were then counted. The SGN density (cells per square millimeter) was calculated using NIH ImageJ 1.43u (Image Processing and Analysis in Java) in a 500 × 500 μM area. The mean number of stained cells was obtained by two researchers blinded to the experimental conditions. SGN count from each group is presented as percentage of survival cells, normalized with control.

### 2.7. Western Immunoblot Analysis

To obtain protein lysates, 12 cochleae of 6 animals/group were pooled and homogenized by using ice cold RIPA buffer (Pierce, Rockford, IL, USA, Cat. No. PI89900). The lysate was sonicated 3 times at 10 Hz (Hielscher, Ultrasound Technology UP50H/UP100H), centrifuged (13,000 rpm, 15 min, 4 °C) and the supernatant (5 µL aliquot) was used to determine the protein concentration (microBCA kit, Cat. No. 23235, Pierce, Rockford, IL, USA). Reducing sample buffer was added to the supernatant, and samples were heated to 95 °C for 5 min. Protein lysates (70 μg) were loaded onto 4–15% Tris-glycine polyacrylamide gels for electrophoretic separation. Precision Plus Protein Dual Color Standards (Bio-Rad) were used as molecular mass standards. Proteins were then transferred onto nitrocellulose membranes at 100 V for 2 h at 4 °C in transfer buffer containing 25 mM Tris (Cat. No. T4661, Sigma-Aldrich, St. Louis, MO, USA), 192 mM glycine (Cat. No. G8898, Sigma-Aldrich), 0.1% SDS (Sodium Dodecyl Sulfate, Cat. No. L3771, Sigma-Aldrich, St. Louis, MO, USA) and 20% methanol (Cat. No. 322415, Sigma-Aldrich, St. Louis, MO, USA). Membranes were incubated for 1 h with blocking buffer (5% skim milk (Cat. No. #1706404, Bio-Rad Laboratories, Hercules, CA, USA) in TBST (Tris Buffered Saline, Cat. No. T5912, Sigma-Aldrich and 0.1% Tween 20, Cat. No. P1379, Sigma-Aldrich, St. Louis, MO, USA), and then incubated overnight at 4 °C with primary antibody against PPARα (1:1000, Cat. No. MA1-822, ThermoFisher, Waltham, MA, USA), PPARγ (1:1000, Cat. No. 2435S, Cell Signaling Tech., Danvers, MA, USA), NF-κB (1:1000, Cat. No. #8242, Cell Signaling Tech., Danvers, MA, USA) and IL-1β (1:100, Cat. No. sc-7884, Santa Cruz Tech., Dallas, TX, USA). After three rinses in TBST (10 min), membranes were incubated for 1 h at RT HRP-conjugated mouse or rabbit secondary antibodies (1:5000; Cat. No. 70765, Cell Signaling, Danvers, MA, USA). Equal protein loading among individual lanes was confirmed by re-probing the membranes with an anti-GAPDH (1:10,000; Cat. No. 9485, Abcam, Cambridge, UK) or anti-α-tubulin mouse monoclonal antibody (1:5000; Cat. No. T5168, Sigma-Aldrich, St. Louis, MO, USA). Protein expression was evaluated and documented by using UVItec Cambridge Alliance. Experiments were performed in triplicate.

### 2.8. Immunofluorescence Analysis on Cochlear Samples

Immunofluorescence analysis were performed on cochlear cryosections 1, 3 and 7 days after the acoustic trauma. As described previously [10,16], 12 cochleae of 6 animals/group were quickly removed and samples were fixed with 4% paraformaldehyde in PBS at 4 °C and pH 7.4. Next, the cochleae were decalcified for 15 days in EDTA (10% EDTA, changed daily), incubated for 48 h in sucrose (30%), embedded in OCT, cryosectioned at a thickness of 12 μM (Cryostat SLEE) and processed for each immunostaining procedure.

#### 2.8.1. DHE Assay

To assess Q-ter and Anakinra protection against noise-induced oxidative damage, we performed immunofluorescence analysis for superoxide anion radicals by using DHE assay.

DHE is a lipophilic cell-permeable dye that is rapidly oxidized to ethidium in the presence of free radicals. In theory, the produced ethidium is fixed by intercalation into nDNA and it gives an indication of cellular oxidant stress [12].

To avoid nonspecific oxidation products and to limit non-specific fluorescence, we acquired images by using two-photon excitation (792 nm, <140 fs, 90 MHz) performed by ultrafast tunable mode locked titanium:sapphire laser (Chameleon; Coherent) coupled to a multiphoton microscope (Nikon Ti-E). Specimens were incubated with 1 mM DHE (Cat. No. D23107, Thermo Fisher, Waltham, MA, USA) in PBS for 30 min at 37 °C and then coverslipped with the antifade medium. Images were taken at 20×.

#### 2.8.2. PPARα and PPARγ Expression in the Cochlear Tissue

In order to detect the expression of PPARα and PPARγ receptors in the cochlea, cryosections were incubated with a blocking solution (1% BSA, 0.5%, Triton X-100 and 10% normal goat serum in PBS 0.1 M) and then they were incubated overnight at 4 °C with a solution containing primary antibody against PPARα (1:100, ThermoFisher) and PPARγ (1:100, Cell Signaling Tech.). All specimens were incubated at room temperature for 2 h in labelled-conjugated donkey anti-mouse and/or anti-rabbit secondary antibody (Alexa Fluor 488 or 546, IgG, Thermo Fisher) diluted 1:400 in 0.1 M PBS and DAPI stained (Cat. No. D1306, Thermo Fisher; 1:1000 in 0.1 M PBS).

#### 2.8.3. Activation of Inflammatory Markers

To perform immunofluorescence analysis on inflammatory markers, cochlear cryosections were incubated with a blocking solution (1% BSA, 0.5%,Triton X-100 and 10% normal goat serum in PBS 0.1 M) and then they were incubated overnight at 4 °C with a solution containing primary antibody against: NF-κB (1:100, Cell Signaling Tech.) and IL-1β (1:100, Santa Cruz Tech.). These antibodies cross-reacted with rat tissue. Cochlear sections were then incubated at room temperature for 2 h in a solution containing donkey anti-rabbit and/or anti-mouse secondary antibodies (Alexa Fluor 488 or 546, IgG, Thermo Fisher) diluted 1:400 in 0.1 M PBS and after stained with DAPI (Cat. No. D1306, Thermo Fisher; 1:500 in 0.1 M PBS) to visualize cell nuclei. For all immunofluorescence analysis, randomly selected sections across experimental groups were used to perform control experiments by omitting the primary antibody. The absence of spontaneous fluorescence in these control sections confirmed the specificity of antibodies (data not shown). Tissues from all groups were always processed together during the procedures to limit variability related to antibody penetration, incubation time, post-sectioning age and condition of tissue. A confocal laser scanning system (Nikon) equipped with an Ar/ArKr laser (for 488 nm excitation) and HeNe laser (for 543 nm excitation) was used to acquire images. DAPI staining was imaged by two-photon excitation (740 nm, <140 fs, 90 MHz) with an ultrafast, tunable mode-locked Ti:sapphire laser.

### 2.9. Statistical Analysis

Power analysis was performed to determine the sample size to provide a statistical power of 80% at an α level of 0.05. The results are presented as mean ± SEM. Statistical analysis was performed by Student’s two-tailed *t*-test. The level of significance was set at 0.05.

## 3. Results

### 3.1. Noise Exposure Induced Functional and Morphological Cochlear Damage

**Figure 2:**
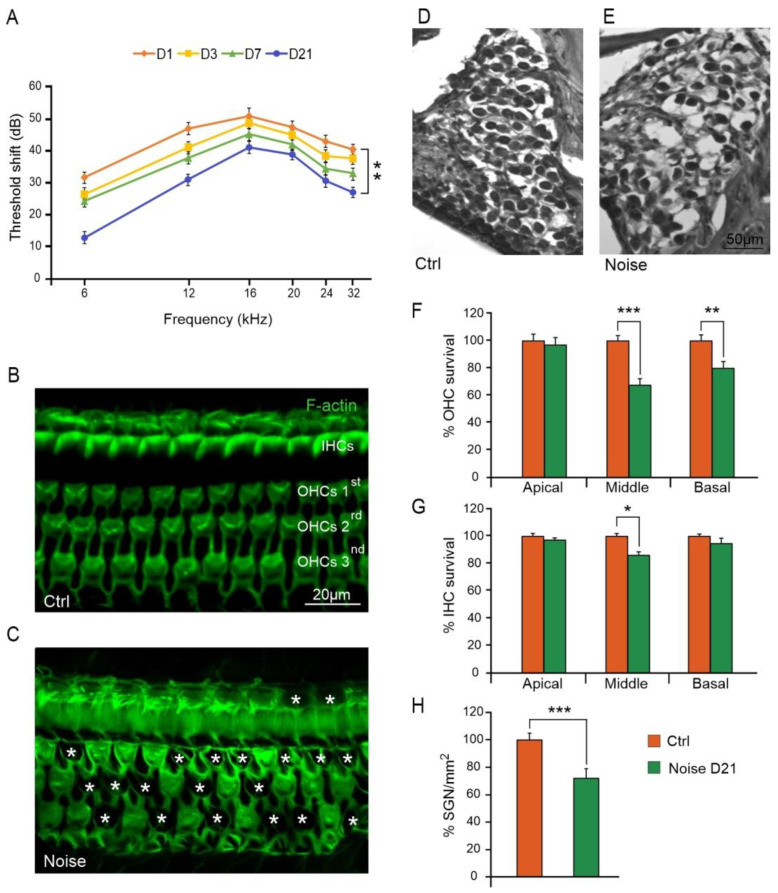
Noise induces functional and morphological cochlear damage. (**A**): Graph (means ± SEM) showing ABR threshold shift values across all frequencies analyzed at 1, 3, 7 and 21 days (D1, D3, D7, D21) after noise exposure. (**B**,**C**): Representative images of surface preparations of the organ of Corti showing the distribution of F-actin in the middle turn region of the cochlea. Normal cochlear organization with three well-aligned rows of outer hair cells (OHCs) and one row of inner hair cells (IHCs) is shown in (**B**) (Ctrl group). In the Noise group, OHC and IHC loss is observed, as indicated by asterisks (**C**). (**D**,**E**): Representative images of spiral ganglion neuron (SGN) cryosections stained with H&E showing a marked reduction in SNG viability in the noise-exposed animals (**E**) compared to control animals (**D**). (**F**,**G**): Histograms (means ± SEM) indicating percentage of OHC and IHC survival in cochlear turns. (**H**): Histograms (means ± SEM) showing percentage of SGN loss in noise-exposed animals compared to control group. Asterisks indicate significant differences between groups (* *p* < 0.05; ** *p* < 0.01; *** *p* < 0.001).

To determine the effect of noise on the auditory function, we recorded ABRs at different time points (1, 3, 7 and 21 days) from the onset of the acoustic trauma. In the Noise group at days 1, 3 and 7, the average threshold shift increased remarkably, reaching about 40–45 dB at 12–32 kHz. At day 21, a permanent threshold shift of about 30–35 dB was still observed (Figure 2A).

To characterize cochlear damage induced by noise exposure, we examined OHC and IHC survival in surface preparations of the organ of Corti. Figure 2 illustrates F-actin staining and hair cell count in Ctrl and Noise groups. In Ctrl animals, OHC and IHC surfaces were characterized by an orderly arrangement of the three rows of OHCs and one row of IHCs (Figure 2B). Noise exposure induced a severe OHC and IHC loss, characterized by dark spots, phalangeal scars and disappearance of both cuticular plates and hair bundles (indicated by asterisks, Figure 2C). In the Noise group, OHC count showed about 80% of hair cell survival in the basal turn and about 65% in the middle turn, as compared to the Ctrl group. No significant differences were observed between groups in the apical turn (Figure 2F). IHC count showed a significant hair cell loss (about 15%) in the middle turn, whereas in the apical and basal turns cell death was less pronounced and not statistically significant (Figure 2G).

We also characterized SGN viability by performing SGN count on cochlear cryosections stained with H&E. In the Ctrl group, the Rosenthal’s canal was densely packed with SGNs and fascicles of auditory nerve fibers (Figure 2D). Twenty-one days after the acoustic trauma, a marked reduction in SGNs was observed (about 30% of SGN loss; Figure 2E,H).

### 3.2. Analysis of Cochlear PPARα and PPARγ Expression after Noise Exposure

**Figure 3:**
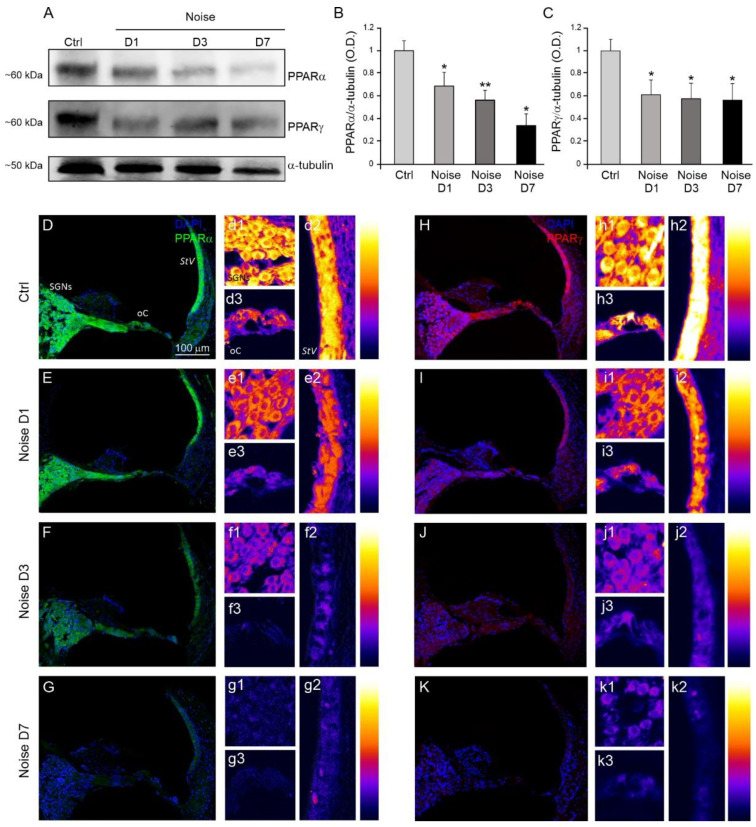
Noise exposure reduces PPARα and PPARγ expression in the cochlea. (**A**): Western immunoblotting showing PPARα and PPARγ reduction at 1, 3 and 7 days (**D**) after noise exposure. (**B**,**C**): Histograms show the decrease in PPARα/α-tubulin and PPARγ/α-tubulin ratios after noise exposure (optical density, O.D.). Data are expressed as mean ± SEM from three independent experiments. Asterisks indicate significant differences between groups (* *p* < 0.05; ** *p* < 0.01). (**D**–**K**): Representative images of cochlear cryosections labeled for PPARα (green fluorescence, (**D**–**G**)) and PPARγ (red fluorescence, (**H**–**K**)) and double-stained with DAPI in blue. Inserts in (**d1**–**k3**) indicate the distribution of fluorescence signal, analyzed in a pseudo-color rainbow scale for the principal cochlear structures. PPARα and PPARγ fluorescence is marked in control samples and progressively decreases from day 1 to day 7 after noise exposure. StV: *stria vascularis*; oC: organ of Corti; SGNs: spiral ganglion neurons. Scale bar: 100 μM.

In order to evaluate the effect of the acoustic trauma on the expression of PPAR receptors, we performed Western blot analysis at different time points (1, 3 and 7 days) from noise exposure (Figure 3). Our results indicate a reduction in PPARα and PPARγ expression in noise-exposed animals, starting from day 1 to day 7, compared to Ctrl animals (Figure 3A–C).

To confirm the Western blot data, we examined PPARα and PPARγ expression by immunofluorescence analysis performed in cochlear cryosections of both Ctrl and Noise samples. We observed a strong fluorescence intensity of both PPARα (green fluorescence) and PPARγ (red fluorescence) signals in Ctrl group (Figure 3D,H). Specifically, PPARα localization was mainly detected in SGNs and *stria vascularis* of Ctrl animals (Figure 3(d1,d2)), whereas a weaker fluorescence signal was found in the organ of Corti (Figure 3(d3)), as indicated by fluorescence readout analysis. Interestingly, in Ctrl animals, immunofluorescence for PPARγ showed a strong signal not only in SGNs and *stria vascularis* (Figure 3(h1,h2)), but also in the organ of Corti (Figure 3(h3)). In particular, a strong immunoreactivity was observed in IHCs with respect to OHCs, suggesting a high expression of this receptor isoform in sensory-neural epithelium.

In the Noise group, we found a progressive decrease in fluorescence intensity for PPARα (Figure 3E–G) and PPARγ (Figure 3I–K) signals, starting from day 1 to day 7 in all cochlear structures.

Collectively, our results reveal that NIHL induces a progressive down-regulation of both PPARα and PPARγ receptors in the cochlea.

### 3.3. Noise Induced Cochlear Inflammatory Response

**Figure 4:**
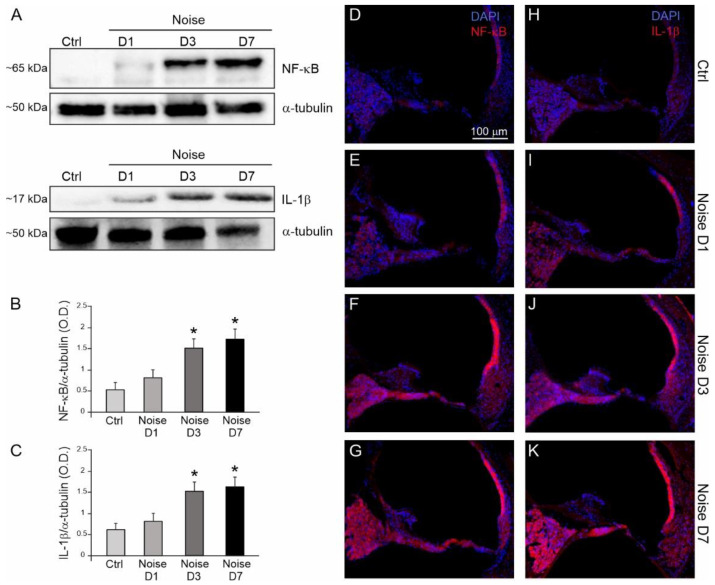
Increased cochlear inflammatory markers after noise exposure. (**A**): Western immunoblotting indicating NF-kB and IL-1β expression levels at 1, 3 and 7 days (D1, D3, D7) after noise exposure with respect to controls. (**B**,**C**): Histograms show the increase in the NF-κB/α-tubulin and IL-1β/α-tubulin ratios after noise exposure (optical density, O.D.). Data are expressed as mean ± SEM from three independent experiments. Asterisks indicate significant differences between groups (* *p* < 0.05. (**D**–**K**): Representative cochlear cryosections labeled with NF-κB (red fluorescence, (**D**–**G**)) and IL-1β (red fluorescence, (**H**–**K**)) and double-stained with DAPI in blue. NF-κB and IL-1β fluorescence is faint in control group (**D**,**H**); NF-κB fluorescence is slight at day 1 after noise exposure (**E**) and increases at day 3 and day 7 (**F**,**G**); IL-1β signal increases progressively from day 1 to day 7 in the Noise group (**I**–**K**). StV: *stria vascularis*; oC: organ of Corti; SGNs: spiral ganglion neurons. Scale bar: 100 μM.

We performed a series of Western blots and immunofluorescence analyses to characterize the expression of cochlear inflammatory markers (NF-κB and IL-1β following noise exposure. Western blot data (Figure 4A–C) showed an increase in NF-κB and IL-1β expression levels. In detail, our results showed a marked increase in NF-κB expression at days 3 and 7, with a starting increase at day 1 (Figure 4A,B); a similar expression pattern was observed for IL-1β (Figure 4A,C).

These results were confirmed by immunofluorescence analysis. In the Ctrl group, fluorescence signal was absent for the inflammatory markers analyzed (Figure 4D,H). Noise exposure induced NF-κB up-regulation, starting from day 1 (Figure 4E), with an increasing trend at day 3 and day 7 (Figure 4F,G). In particular, a strong fluorescence signal was observed in the SGNs and especially in the *stria vascularis* of noise-exposed animals; a minor fluorescence increase was found in the organ of Corti. These results are in line with previous data showing that NF-κB expression involved mainly SGNs and *stria vascularis* in a model of cochlear damage induced by noise [18]. Increasing IL-1β signal was observed after noise exposure at day 1 (Figure 4I) and day 3 (Figure 4J), reaching the highest expression at day 7 (Figure 4K).

Taken together, immunofluorescence data show a strong activation of inflammatory processes, in agreement with Western blot results.

### 3.4. The Effects of Q-Ter and Anakinra on PPAR Expression, Oxidative Damage and Inflammatory Processes

**Figure 5:**
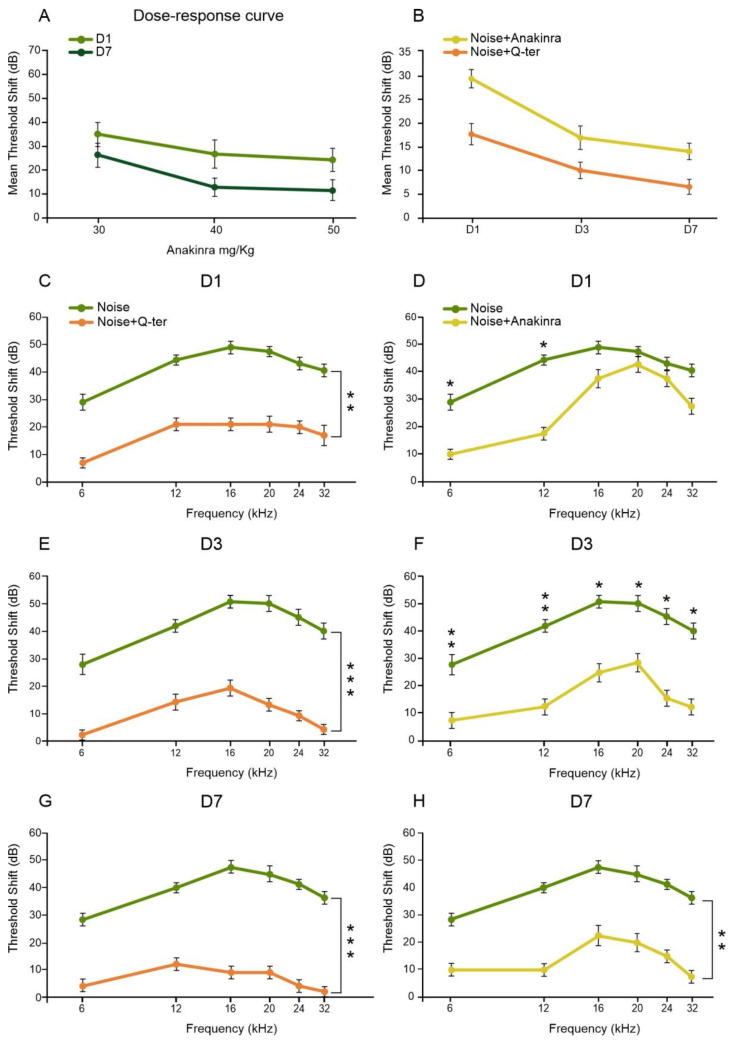
Protective effects of Q-ter or Anakinra treatments against noise-induced hearing loss. (**A**): Dose–response curve showing the best auditory protection obtained with a dosage of 40 mg/kg of Anakinra. (**B**): Mean auditory threshold shift in Noise+Qter and Noise+Anakinra groups measured at 1, 3 and 7 days (**D1**,**D3**,**D7**) after trauma. Notably, Q-ter treatment shows an earlier protective effect on hearing function. (**C**–**H**): Threshold shift values (mean ± SEM) showing the protective effect of Q-ter (**C**,**E**,**G**) and Anakinra (**D**,**F**,**H**) at different time points after noise exposure. Q-ter attenuates threshold shift by about 20 dB at day 1 (D1) after noise exposure (**C**), about 25 dB at day 3 (**D3**, **E**) and about 30 dB at day 7 (**D7**, **G**) after noise exposure. The protective effect of Anakinra is delayed with respect to Q-ter; the best protective effect is observed after 7 days from acoustic trauma, with a threshold shift attenuation of about 20 dB (**H**). Asterisks indicate significant differences between groups (* *p* < 0.05; ** *p* < 0.01; *** *p* < 0.001).

Given that PPAR nuclear receptors play a critical role in the transcriptional modulation of various cellular functions, such as regulation of glucose and lipid metabolism, cell growth, differentiation and inflammation [35,36], they can be considered important “sensors” of various metabolic and inflammatory stimuli. Therefore, looking for the molecular mechanisms underlying PPAR down-regulation induced by noise exposure, we used an antioxidant (Q-ter) and anti-inflammatory (Anakinra) treatment to study the role of a metabolic or inflammatory insult in PPAR cochlear expression. Thus, we first analyzed the protective effect of Q-ter or Anakinra supplementations on auditory function. We recorded ABRs in all experimental groups at 1, 3 and 7 days after noise exposure. A dose–response curve was obtained at days 1 and 7 by using different doses of Anakinra (30, 40 and 50 mg/kg). The curve obtained in the 15 animals of the preliminary study revealed that the best auditory protection was obtained with 40 mg/kg of Anakinra (Figure 5A). Ctrl animals treated with Q-ter and Anakinra alone showed no significant differences compared to Ctrl animals (data not shown). Animals treated with Q-ter showed a threshold shift attenuation for all frequencies, as compared with the Noise group, at all time points (Figure 5C,E,G). Specifically, at day 1 and day 3, Q-ter attenuated the threshold shift of about 20 dB and 25 dB, respectively (Figure 5C,E). Moreover, at day 7, a further attenuation (by about 10 dB) was observed (Figure 5G). In regard to Anakinra supplementation, we observed the most protective effect of the anti-inflammatory treatment after 3 and 7 days from the acoustic trauma, when threshold shift in Noise+Anakinra group was about 20–25 dB, spanning all frequencies (Figure 5F,H). Of note, comparing the effect of Q-ter and Anakinra supplementation, the anti-inflammatory treatment showed a delayed otoprotection, starting from 3 days after noise insult, whereas the antioxidant treatment showed an earlier protective effect, starting from 1 day after the acoustic trauma as shown in Figure 5B.

**Figure 6:**
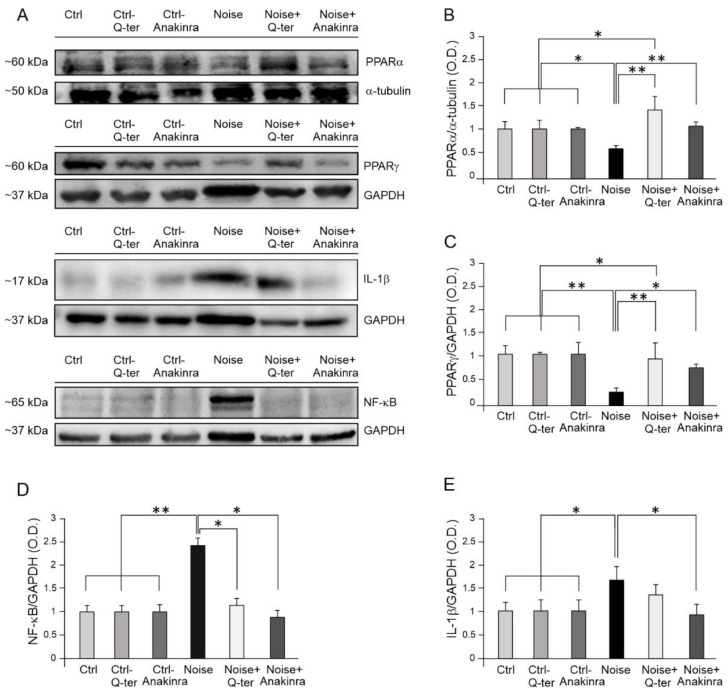
Cochlear PPAR and inflammatory marker expression in Q-ter and Anakinra-treated samples. (**A**): Western immunoblotting showing cochlear expression of PPARα, PPARγ, NF-κB and IL-1β in animals exposed to noise and treated with Q-ter or Anakinra (day 7 after acoustic trauma). (**B**–**E**): Histograms showing densitometry evaluations (O.D., proteins/GAPDH ratios). Data are expressed as mean ± SEM from three independent experiments. Asterisks indicate significant differences between groups (* *p* < 0.05; ** *p* < 0.01).

Furthermore, with the overall goal to analyze in depth the effect of an antioxidant and/or an anti-inflammatory treatment on the regulation of PPAR receptors in response to noise exposure, we performed a Western blot analysis at day 7 after acoustic trauma for PPARα and PPARγ (Figure 6). Our results revealed that Q-ter treatment has a major effect in preventing PPARα and PPARγ down-regulation following noise exposure, with respect to Anakinra treatment (Figure 6A–C). Specifically, Q-ter treatment was able to increase PPARα cochlear levels over the basal condition, probably potentiating antioxidant PPARα activity against redox unbalance (Figure 6A,B). PPARγ expression also increased markedly after antioxidant supplementation, returning to control levels (Figure 6A,C). On the other hand, Anakinra treatment restored basal level of PPARα (Figure 6A,B), but, despite increasing PPARγ expression with respect to Noise condition, Anakinra was not able to reestablish PPARγ to the control level (Figure 6A,C).

**Figure 7:**
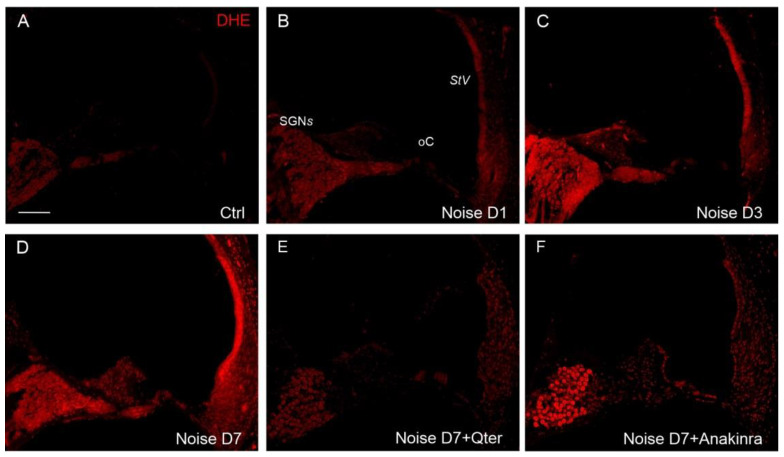
Cochlear ROS amount in Q-ter and Anakinra treated animals. Representative images of cochlear cryosections stained with DHE (red fluorescence). Superoxide fluorescence is faint in the control group (**A**). In the Noise group, DHE fluorescence progressively increases from day 1 to day 7 D1–D7) in all cochlear structures (**B**–**D**). Q-ter supplementation decreases superoxide expression in all cochlear structures (*stria vascularis*, organ of Corti and SGNs) (**E**), whereas Anakinra treatment attenuates ROS amount in the organ of Corti and *stria vascularis*, with a poor protective effect in SGNs (**F**). StV: *stria vascularis*; oC: organ of Corti; SGNs: spiral ganglion neurons. Scale bar: 100 μM.

By using DHE assay, we evaluated the effect of drug otoprotection on ROS cochlear amount. As expected, noise induced a progressive increase in ROS production from day 1 to day 7 (Figure 7B–D), consistent with previous studies [16,18]. Q-ter treatment significantly decreased ROS amount in all cochlear structures (Figure 7E), specifically in SGNs, suggesting that neural tissue could be more susceptible to oxidative stress. Anakinra supplementation attenuated ROS amount specifically in the organ of Corti and *stria vascularis* (Figure 7F), whereas a high fluorescence intensity signal was still observed in SGNs.

Furthermore, considering that Anakinra neutralizes the biological activity of IL-1α and β by competitively inhibiting the two cytokines binding to the IL-1 receptor [37,38], we gained insights into the downstream molecular pathway by analyzing the expression of the key inflammatory mediators following Anakinra treatment. Indeed, as expected, Western blot results showed that Anakinra induced a down-regulation of IL-1β probably responsible for NF-κB reduction (Figure 6A,D,E). Similarly, we observed a significant reduction in NF-κB expression in noise-exposed animals treated with Q-ter with respect to the Noise group; however, this effect was not associated with a down-regulation of the pro-inflammatory cytokine IL-1β (Figure 6A,D).

Collectively, our functional and molecular data show that oxidative stress is the primary element of damage in noise-induced hearing loss and it can be speculated that increased inflammation can be considered a consequence of PPAR down-regulation induced by ROS production. Indeed, Q-ter treatment attenuated auditory threshold increase induced by noise already at 1 day after acoustic trauma. This earlier protective effect of Q-ter with respect to Anakinra treatment leads us to consider ROS production responsible for PPAR decreased expression and, consequently, increased inflammation.

## 4. Discussion

The purpose of our study was to evaluate the role of oxidative stress and inflammatory processes in mediating noise-induced cochlear damage, focusing on PPAR receptor signaling. To this aim, we used an antioxidant (Q-ter) and anti-inflammatory (Anakinra) treatment as a tool to investigate how decreasing oxidative stress or inflammatory responses could modulate PPAR expression, attenuating hearing loss. Our results show that (1) noise exposure induces a progressive decrease over time in both PPARγ and PPARα expression in cochlear structures; (2) reduced expression of PPARs is associated with oxidative stress and a progressive increase in inflammatory markers (NF-κB and IL-1β) in noise-exposed cochleae; (3) blocking oxidative stress through an antioxidant treatment reestablishes basal PPAR levels in the cochlea, leading to decreased expression of the redox-sensitive transcription factor of inflammation related genes NF-κB; (4) blocking inflammation, both IL-1β and NF-κB decreased, leading to a ROS reduction in the organ of Corti and *stria vascularis*.

**Figure 8:**
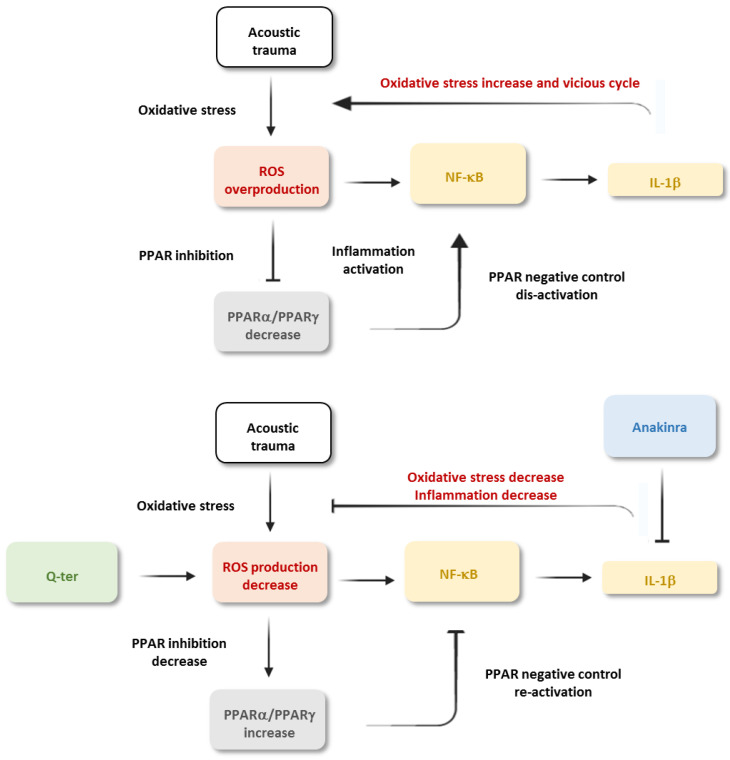
Suggested mechanisms of damage in noise-induced cochlear injury. Acoustic trauma induces an increase in ROS amount in the cochlea, leading to oxidative stress and inflammation. Noise-induce metabolic damage can affect PPAR cochlear expression, leading to a down-regulation of PPAR receptors that, in turn, is responsible for the dis-activation of the negative control on inflammatory signaling. Indeed, this could lead to an increase in inflammatory markers, pro-inflammatory cytokines (IL-1β) and NF-κB. Given the interplay between oxidative stress and inflammation, the rise in inflammatory markers could exacerbate oxidative stress, causing a damaging vicious cycle among oxidative stress, PPAR down-regulation and inflammation. An antioxidant treatment with Q-ter can stop the feedback loop by blocking oxidative stress, the key damaging player in cochlear injury induced by noise. In our hypothesis, decreased ROS production consequently could decrease PPAR inhibition inducing PPAR negative control re-activation on inflammatory markers. An anti-inflammatory treatment (Anakinra) shows less protective effects.

Altogether, on the basis of our findings, we can speculate that oxidative stress induced by noise exposure caused a PPAR down-regulation, with a consequent dis-activation of PPAR negative control on inflammatory markers. On the other hand, decreasing oxidative stress with Q-ter increased PPAR level and reactivated PPAR negative control of inflammatory markers (Figure 8).

Notwithstanding the emerging role of PPARs as therapeutic targets against cochlear injury, no previous reports have described the expression of PPARs in the inner ear in a in vivo model of NIHL. By Western blot and immunofluorescence analysis, we showed that PPARα and PPARγ cochlear expression was severely affected by noise exposure, with a progressive decrease in protein levels over time from day 1 to day 7 after acoustic trauma.

Looking for a molecular mechanism underlying PPAR down-regulation induced by noise, we focused on cochlear oxidative stress and inflammatory mediators.

In the cochlea, the role of oxidative stress in damage initiation and progression has been supported by the generation of ROS in cochlear tissues observed at very early time points (7 h after exposure to damaging levels of noise) [39] and persisting for 7–10 days after noise exposure [40,41]. Consistent with literature data and our previous published results [16,18,42], we found an early and progressive increase in ROS production in all cochlear structures, reaching a maximum expression 7 days after noise insult. This increase in ROS production could be related to observed PPAR cochlear down-regulation. Indeed, PPAR expression and activity may be altered by cellular energy metabolism status; in fact, oxidative stress regulates a variety of signaling pathways that subsequently affect gene expression by modulating a large number of transcription factors, including PPARs. It has been shown that hydrogen peroxide induced-oxidative stress in vascular endothelial cells attenuates PPARγ expression and activity through suppression of PPARγ transcription, potentially via activating inhibitory redox-regulated transcription factors [43]. At the same time, antioxidant supplementation with vitamin E can effectively restore PPARα expression in aged mice to levels seen in younger mice [44].

By analyzing cochlear inflammatory responses, we also found an up-regulation, between 1 and 7 days after trauma, of the principal inflammatory markers (NF-κB and IL-1β), in conjunction with the decreased expression of PPARs. Several inflammation-related genes and proteins have been implicated in cochlear response both to acute noise exposure [6,45,46,47] and chronic environmental noise exposure [8]. Cumulative evidence also indicate an interplay between ROS and inflammation in noise trauma, ototoxicity and age-related hearing loss [5,10,14,47,48,49,50,51,52]. Moreover, several studies have demonstrated PPAR ability to inhibit the expression of inflammatory markers and signaling pathways, such as cytokines, metalloproteases and acute phase proteins [53,54,55,56,57]. In order to establish a cause-effect relationship among PPAR down-regulation, increased oxidative stress and increased inflammatory markers in cochlear noise-induced injury, we used both antioxidant (Q-ter) and anti-inflammatory (Anakinra) treatments for investigating the effect of blocking oxidative stress or direct inhibiting inflammation. Q-ter is a water soluble CoQ10 analogue that shows enhanced bioavailability with respect to the native form [58]. We previously reported the efficacy of Q-ter administration in counteracting oxidative damage in cochlear insult induced by noise or ototoxic drugs [12,13,34,59]. Indeed, here, Q-ter administration showed early protective effects against noise damage, attenuating hearing loss of about 30 dB, since day 1 after noise exposure. As an anti-inflammatory drug, we choose Anakinra, a recombinant form of IL-1R used to treat a broad variety of diseases, ranging from common conditions, such as rheumatoid arthritis, gout and idiopathic pericarditis, to rare hereditary diseases [60]. Our data revealed that Anakinra showed protective effects on hearing function starting from day 3 after noise exposure. Considering that Anakinra is the recombinant form of the naturally occurring IL-1 receptor antagonist, our functional data are consistent with Western blot analysis, showing a significant increase in IL-1β cochlear expression starting 3 days after noise exposure. The effect of Anakinra in reducing IL-1β activation also resulted in decreasing NF-κB cochlear levels, according to literature data showing that IL-1 activation leads to phosphorylation and degradation of IκB proteins, releasing NF-κB and allowing its translocation to the nucleus, where it controls the expression of downstream pro-inflammatory genes [61,62]. Moreover, the efficacy of Anakinra treatment in decreasing both IL-1 and NF-κB in several disease models has been documented [63,64,65]. Our data showing decreased expression of cochlear inflammatory markers after antioxidant Q-ter treatment support the hypothesis of a cross-talk between oxidative and inflammatory damage and NF-κB plays a key role in mediating this interplay. Indeed, ROS interact with the NF-κB signaling pathway in many ways [1,2,3] and the transcription of NF-κB-dependent genes has been shown to influence the levels of ROS in the cell and, in turn, the levels of NF-κB activity are also influenced by the levels of ROS [1,42,66,67].

Interestingly, after Q-ter treatment, PPAR level returned similar to control values. In particular, expression of PPARα is potentiated, with higher levels compared to control ones, whereas PPARγ expression returns to basal level. Anakinra treatment induced a significant increase in PPAR expression with respect to noise condition, although to a lesser extent compared to Q-ter effect. Thus, comparing antioxidant and anti-inflammatory treatment, the major effect in reestablishing and/or potentiating PPAR levels was observed after Q-ter treatment. This leads us to establish a relation between increased oxidative stress and decreased PPAR expression and, in turn, it is associated with increased inflammation, with a vicious cycle between oxidative stress, inflammatory responses and PPAR down-regulation in mediating cochlear damage. In fact, much evidence has shown that metabolic stressors can modulate PPAR expression [68,69,70,71,72]. Moreover, we previously showed that noise induced a down-regulation of Nrf2 pathway, exacerbating redox imbalance between increased ROS production and decreased endogenous antioxidant ability [16,42]. Interestingly, studies have strongly suggested existence of reciprocal regulation of Nrf2, a transcription factor involved in the endogenous antioxidant response, and PPAR pathways to reinforce the expression of one another [73,74,75].

## 5. Conclusions

Taken together, our data showed that noise-induced cochlear damage involves PPARs down-regulation, caused mainly by redox status imbalance. In fact, ROS production can lead to increased inflammatory markers, both through PPAR modulation and through ROS/inflammation interplay, leading to a positive loop between cochlear oxidative stress/inflammatory responses and PPAR down-regulation that, in turn, negatively modulates cochlear endogenous antioxidant and anti-inflammatory response to damage.

On a translational perspective, our data support the key role of oxidative stress in noise-induced cochlear damage, suggesting that early targeting of oxidative stress to reestablish PPAR signaling pathways could represent the best strategy to restore hearing loss by blocking, at the same time, the inflammatory response caused by metabolic damage.

## Data Availability

Data that support the findings of this study are available from the corresponding author on reasonable request.
